# A multicenter observational retrospective study of second-line treatment with daratumumab–bortezomib–dexamethasone (DaraVd) in multiple myeloma patients refractory to lenalidomide

**DOI:** 10.1007/s10238-025-01956-w

**Published:** 2025-11-25

**Authors:** I. Rizzello, I. Sacchetti, S. Barbato, V. Solli, P. Stefanoni, L. Cani, M. Quaresima, A. Belotti, N. Sgherza, M. Gentile, R. Zambello, G. Barilà, M. Celli, G. Mele, K. Mancuso, L. Pantani, P. Tacchetti, M. Talarico, M. Puppi, E. Manzato, R. Restuccia, S. Masci, S. Ghibellini, F. Bigi, M. Cavo, E. Zamagni

**Affiliations:** 1 IRCCS Azienda Ospedaliero-Universitaria Di Bologna, Istituto Di Ematologia “Seràgnoli”, Bologna, Italy; 2 Dipartimento Di Scienze Mediche E Chirurgiche, Università Di Bologna, Bologna, Italy; 3 Department of Oncology and Hematology, ASST Papa Giovanni XXIII, Bergamo, Italy; 4 Division of Hematology, AOU Città Della Salute e Della Scienza di Torino, Turin, Italy; 5 Azienda USL-IRCCS Di Reggio Emilia, Reggio Emilia, Italy; 6 Department of Hematology, ASST Spedali Civili di Brescia, Brescia, Italy; 7 Hematology and Stem Cell Transplantation Unit, AOU Consorziale Policlinico, Bari, Italy; 8 Hematology Unit AO of Cosenza, Cosenza, Italy; 9 Department of Pharmacy, Health and Nutritional Sciences, University of Calabria, Rende, Italy; 10 Dipartimento Di Medicina (DIMED), Unità di Ematologia, Università di Padova, Padua, Italy; 11 Hematology Unit, San Bortolo Hospital, Vicenza, Italy; 12 UO di Ematologia, Ospedale Infermi, Rimini, Italy; 13 U.O.C. di Ematologia - Unità Trapianto di Midollo Osseo; P.O. Antonio Perrino, 72100 Brindisi, Italy

**Keywords:** Multiple myeloma, Relapsed/refractory multiple myeloma, Lenalidomide-refractory, Second-line DaraVd, Real-life DaraVd

## Abstract

Upfront lenalidomide in newly diagnosed multiple myeloma has become the gold standard. Nonetheless, proper management of lenalidomide-refractory patients is challenging. Daratumumab–bortezomib–dexamethasone (DaraVd) has been approved after ≥ 1 prior therapy, but data on upfront lenalidomide refractoriness are scarce. We run a retrospective study to assess the outcomes of 85 lenalidomide-refractory patients treated at first relapse with DaraVd in 10 Italian centers. Baseline characteristics were representative of a general population, despite inferior median age (57 years). Sixteen (27%) at diagnosis and 7 (29%) at relapse had high-risk cytogenetics (t(4;14) and/or t(14;16) and/or del17). Furthermore, one had del1p, one gain1q (3 copies) and one amp1q (≥ 4 copies) at diagnosis; one gain1q and one amp1q at relapse. Overall response rate was 86% (61% ≥ VGPR). Median PFS and OS were 15 and 47 months, respectively (25-months median follow-up). Previous dose/duration of lenalidomide did not influence PFS, favorably affected by the absence of amp1q (*p* = 0.04), bone marrow plasma cells < 60% (*p* = 0.003), absence of extramedullary-disease (*p* = 0.009), and ≥ VGPR (*p* < 0.001) or ≥ CR (*p* = 0.012). In a multivariate model, response ≥ VGPR was confirmed to be independently associated to PFS (median: 26 vs. 7 months). At least one grade ≥ 2 adverse event (AE) occurred in 67 (85%) patients. Most common AEs were hematological (72%), infections (30%, 8% grade-3, 1% grade-5), pneumonia (14%), and asthenia (38%). Peripheral neuropathy rate was 58% (8% grade-3). Toxicity-related bortezomib dose reduction occurred in 39 (49.4%) patients; 27 (44%) delayed Dara (1 median dose), mostly for infections. Three patients discontinued for toxicity. Collectively, second-line DaraVd remains an alternative in lenalidomide-refractory patients, especially for those ineligible for pomalidomide/carfilzomib-based regimens, T-cell-redirecting therapies or other experimental drugs.

## Highlights

- Proper management of multiple myeloma (MM) patients relapsing on upfront lenalidomide is challenging.
- Daratumumab-bortezomib-dexamethasone (DaraVd) is approved after ≥1 prior therapy in MM.
- Data on second-line DaraVd in lenalidomide-refractory MM patients are scarce.
- A retrospective study on 85 patients confirmed DaraVd as a viable option in this setting.

## Introduction

Over the last two decades, the treatment paradigm for multiple myeloma (MM) has undergone rapid and continuous evolution, leading to dramatical improvements and prolonged survival rates [1]. In particular, the advent of lenalidomide-based triplets has led to significant improvement in the outcomes of newly diagnosed (ND) and relapsed/refractory (RR) MM patients [2–6].

In the current therapeutic scenario, the gold standard treatment for most of NDMM patients, both eligible or not for autologous stem cell transplantation (ASCT), includes lenalidomide (len) as continuous treatment until progression [7, 8]. At the time of first, and subsequent, relapse(s), the treatment is guided by any acquired refractoriness to previous drugs, age and performance status of the patient, presence of any toxicities, and aggressiveness of the disease. Historically, first choice for second-line therapy included len-based triplets (i.e., carfilzomib–lenalidomide–dexamethasone [KRd], daratumumab–lenalidomide–dexamethasone [DaraRd], ixazomib–lenalidomide–dexamethasone [IRd], or elotuzumab–lenalidomide–dexamethasone [EloRd]) [2–5]. However, the extensive use of len as upfront strategies is progressively expanding the number of len-refractory (len-ref) patients already at first relapse, making it increasingly difficult to manage these patients, who currently have suboptimal outcomes, and to identify the most appropriate therapy in this setting [9].

At present, according to the European Medicines Agency (EMA) approval, currently approved regimens for patients progressing under upfront len-based therapies are represented by: daratumumab and bortezomib plus dexamethasone (DaraVd), carfilzomib plus dexamethasone (Kd), pomalidomide and bortezomib plus dexamethasone (PVd), daratumumab and pomalidomide plus dexamethasone (DaraPd), isatuximab and carfilzomib plus dexamethasone (IsaKd), daratumumab plus carfilzomib and dexamethasone (DaraKd), selinexor and bortezomib plus dexamethasone (SelVd), and cilta-cel (ciltacabtagene autoleucel) [10–17]. More recently, combinations with belantamab mafodotin plus either bortezomib and dexamethasone (BelaVd) or pomalidomide and dexamethasone (BelaPd) received a positive opinion from EMA Committee for Medicinal Products for Human Use (CHMP), though not approved by EMA at the moment of the writing of this paper [18], [19].

All these therapeutic options are characterized by a wide range of efficacy and different toxicity profiles. Briefly, regarding efficacy, the median (m) progression-free survival (PFS) rates reported with these combinations in the whole patient populations enrolled into the registrational trials were: 11.2 months for PVd (OPTIMISMM trial) 12.4 months for DaraPd (APOLLO trial), 13.9 months for SelVd (BOSTON trial), 16.7 months for DaraVd (CASTOR trial), 18.7 months for Kd (ENDEAVOR trial), 35.7 months for IsaKd (IKEMA trial), 28.4 months for DaraKd (CANDOR trial), not reached (NR) for cilta-cel (CARTITUDE-4 trial, with a 12-month PFS of 75.9%), 36.6 months for BelaVd (DREAMM-7 trial), NR for BelaPd (DREAMM-8 trial, with a 12 months PFS of 71%) [10–19]. Of note, while the OPTIMISMM, APOLLO, and DREAMM-8 trials included 100% of len-exposed and high percentages of len-ref patients (71% for PVd, 23% of whom at first relapse, 79% for DaraPd, and 81% for BelaPd) [13, 14, 19], and the CARTITUDE-4 trial was subsequently designed for len-ref patients (enrolling 32.7% of len-ref at first relapse) 17, these patients were underrepresented in the ENDEAVOR, IKEMA, CANDOR, BOSTON, and DREAMM-7 trials, in which only 24%, 32%, 32%, 27%, and 34% of len-ref patients were included, respectively [10, 12, 15, 16, 18]. Similarly, the CASTOR trial enrolled 24% of len-ref patients (only 5% relapsed on first-line len), showing superior efficacy of DaraVd over bortezomib plus dexamethasone (Vd), and culminating in the approval of the triplet combination after at least 1 prior therapy [20]. Since then, DaraVd has been widely used, especially until the subsequent advent of more active combinations; nonetheless, few data have been collected on its use as second-line therapy in len-ref patients so far. To amend this lack of knowledge, a retrospective observational study was designed to explore the use of DaraVd in len-ref patient at first relapse, as its efficacy and safety in this specific subgroup of patients were not described in the CASTOR trial, nor in other real-life experiences.

## Materials and methods

### Study design and patients

This was a multicentric, observational, retrospective study conducted in 10 Italian hematologic sites. Eligible patients were aged 18 years and older and had a measurable, secretory, active (according to CRAB and new biomarkers of malignancy) MM [21]. As per inclusion criteria, enrolled patients had to be first-time relapsers and refractory to len (i.e., patients progressing during treatment or within 60 days after stopping therapy). To be included in the present study, patients were treated with DaraVd outside clinical trials between June 2018 and December 2022, for at least two cycles after first relapse. The study was approved by Comitato Etico Area Vasta Emilia Romagna (CE-AVEC) and by local Ethics Committees at each participating site and was conducted in accordance with the International Conference on Harmonization Guidelines on Good Clinical Practice and the principles of the Declaration of Helsinki. All participants signed an informed consent form prior to their inclusion in the study.

### Objectives of the study

The primary objective was to evaluate the PFS in len-ref patients treated with second-line DaraVd in real-life. Secondary endpoints aimed at collecting data on hematological response rates, overall survival (OS), time to next treatment (TTNT), second PFS (PFS2), and at exploring possible correlations between response rates and survival outcomes with baseline patient characteristics and risk factors. Also, the safety profile of this triplet regimen in real life was explored.

### Statistical analysis

All consecutive patients referred to the study centers who met all the eligibility criteria were enrolled in the study and registered in an e-CRF web-based database. Descriptive statistics were presented for baseline characteristics; continuous variables were summarized by mean and standard deviation or median, min, max, and interquartile range (IQR), as appropriate. Categorical variables were summarized by using absolute and relative frequency distribution. The treatment regimens used, in terms of drug dose, duration of treatment, interruptions, dose reductions, and concomitant therapies, were presented by means of descriptive statistics as well. Response to treatment was evaluated according to the International Myeloma Working Group [IMWG] Uniform Response Criteria for MM [22]. The response distribution was then reported together with the overall response rate (ORR), defined as the proportion of patients who achieved at least a partial response to therapy.

Time-to-event endpoints were calculated from the start of the induction treatment date and analyzed according to the Kaplan-Meier (KM) method. PFS and PFS2 were defined as the time from start to therapy to the first (PFS) or second (PFS2) disease progression or death, whichever came first; TTNT was defined as the time from the start of therapy to the start of the subsequent line of therapy. OS was the time to death. Factors independently affecting PFS and OS were evaluated with a multivariable Cox regression model fitted with still significant covariates in the previous univariate regression analysis.

Toxicity was expressed in terms of adverse events (AEs) occurring before, during, or after treatment and presented by means of descriptive statistics. AEs were evaluated as per the Common Terminology Criteria for Adverse Events (CTCAE, version 5).

All analyses were performed using R version 4.4.1, a statistical computing language and environment developed by the R Foundation for Statistical Computing (Vienna, Austria). Two-sided p values were used, and 0.05 was taken as the cutoff for statistical significance. All confidence intervals (CI) were reported as 95% CI.

## Results

### Patients’ characteristics

Eighty-five patients treated with DaraVd outside clinical trials in 10 Italian hematologic centers were enrolled from June 2018 to December 2022 and included in the analysis. Forty-four percent of the patients were male. At the time of initiation of treatment with DaraVd, the median age was 68 years old; 19% of the patients were aged between 65 and 70 years and 45% were older than 70. The most frequent MM isotype was IgG, in 60% of the patients; other isotypes were IgA in 18% and IgD in 1%, while 21% had a Bence-Jones MM. Median creatinine was 0.85 mg/dL (IQR 0.72–1.01), and median serum calcium was 9.1 mg/dL (IQR 8.8–9.6). In evaluable patients (at diagnosis: 83 for the International Staging System, ISS, and 61 for the revised ISS, R-ISS), ISS stages 1, 2, and 3 were 47%, 33%, and 19%, respectively, and R-ISS stages 1, 2, and 3 were 36%, 56%, and 8%, respectively. Fluorescence in situ hybridization (FISH) at diagnosis showed a high cytogenetic risk in 16 out of 59 evaluable patients, due to the presence of deletion (del) of chromosome 17 and/or t(4;14) and/or t(14;16), while 44 patients were considered to be standard risk. Regarding chromosome 1, one patient had del1p, one had gain1q (3 copies), and one amp1q (≥ 4 copies). At first relapse, FISH was evaluable in 24 patients only: a high cytogenetic risk was recorded in 14 patients, while 10 were considered to be at standard risk, one patient had gain1q and one amp1q. Extramedullary disease (EMD), evaluated by PET/TC FDG, was recorded in 5 of 68 evaluable patients.

Among the enrolled patients, first-line therapies included bortezomib-based triplets in 40 (47%) of them, while 35 patients (41%) received a len-based treatment (mostly len-dexamethasone as continuous therapy), and 10 (12%) received a carfilzomib-based triplet. Collectively, 47 patients received single (n = 26) or double (n = 21) ASCT: allocation to single or double transplantation was at the discretion of the physician or based on center policy. No patients were refractory to bortezomib, and one patient only was refractory to carfilzomib. All the patients received len in first line, either as continuous treatment or as maintenance therapy after ASCT. Median time of len exposure in first line was 12.5 months (range 2-95 months). Len dose at the time of relapse was 10 mg in about half of the patients (n = 47), 25 mg in 16 patients, 15 mg/die or 20 mg/die in 8 patients, and less than 10 mg/die in 10 patients. Median PFS with first-line treatment was 27 months, being 27 months in patients treated with ASCT and 25 months in transplant-ineligible patients.

After first relapse, all patients received DaraVd as second-line therapy at a median of 28 months from the diagnosis. Specifically, 3 patients received daratumumab subcutaneously (SC), while 82 started with intravenous (IV) administration—as SC formulation was not approved at the time of treatment initiation—and then switched to SC route of administration, when available. All patients started with full dose of bortezomib (1.3 mg/m^2^), except for 3 patients who started with reduced dose (1 mg/m^2^ or 0.7 mg/m^2^) because of pre-existing grade-1 or grade-2 peripheral neuropathy (PN). Only 27 patients started with dexamethasone at full dose as for approved schedule (20 mg at days 1–2-4–5-8–9-11–12/21). Collectively, 3 patients received salvage ASCT during DaraVd treatment, after 5 (n = 2) or 14 (n = 1) cycles of induction, and 2 of them continued DaraVd treatment after transplantation.

Patient characteristics are described in Table 1.

**Table 1:** Baseline Characteristics

Characteristics	Number (%)
Age at relapse, median (years)	68
< 65 years	31 (36)
65–70 years	16 (19)
> 70 years	38 (45)
*Sex*	
Male	37 (44)
Female	48 (56)
*Isotype*	
IgG	51 (60)
IgA	15 (18)
IgD	1 (1)
BJ	18 (21)
Creatinine at relapse (mg/dL) (median) [IQR]	0.85 [0.72–1.01]
Calcium at relapse (mg/dL) (median) [IQR]	9.1 [8.8–9.6]
*Medullary PCs at relapse*	
< 60%	28 (33)
≥ 60%	9 (11)
NA	48 (56)
*ISS*	
I	39 (46)
II	28 (33)
III	16 (19)
NA	2 (2)
*R-ISS*	
I	22 (26)
II	34 (40)
III	5 (6)
NA	24 (28)
*Cytogenetic risk at diagnosis*	
Hi-R	16 (19)
St-R	44 (52)
NA	25 (29)
EMD at relapse	5 (6)
Prior ASCT	47 (55)
Single	26 (30)
Double	21 (25)
Bortezomib exposed	40 (47)
Carfilzomib exposed	10 (12)
Median time of lenalidomide exposure [IQR]	12.5 [7.25–22.75]
*Lenalidomide dose at relapse (mg)*	
25 – 15	24 (28)
10 – 2.5	57 (67)
NA	4 (5)

Abbreviation: ASCT, autologous stem cell transplantation; BJ, Bence-Jones; EMD, extramedullary disease; Hi-R, high-risk (defined as the presence of deletion of chromosome 17 and/or t(4;14) and/or t(14;16)); IQR, interquartile range; ISS, international staging system; NA, not available; PCs, plasma cells; R-ISS, revised international staging system; St-R, standard risk (defined as the absence of deletion of chromosome 17 and/or t(4;14) and/or t(14;16))

### Response and survival outcomes

**Fig. 1:**
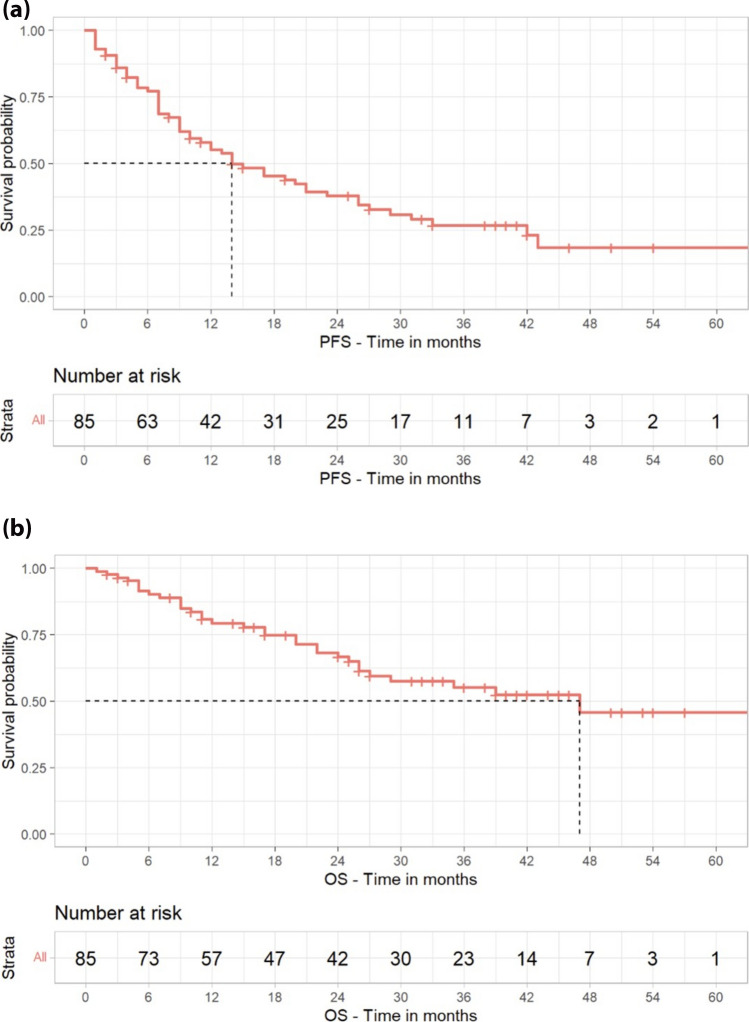
**(a)** Progression-free survival of the whole population. Abbreviation: PFS = progression-free survival. **(b)** Overall Survival of the whole population. Abbreviation: OS = overall survival

**Fig. 2:**
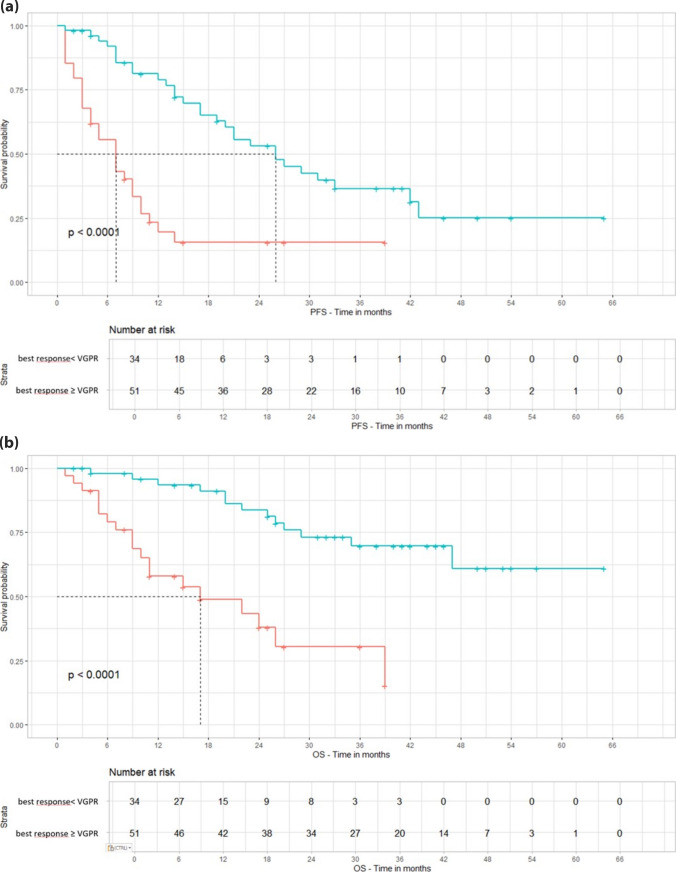
**(a)** PFS stratified by response < or ≥ very good partial response. Abbreviation: PFS = progression-free survival; VGPR = very good partial response. **(b)** Overall Survival stratified by response < or ≥ very good partial response. Abbreviation: OS = overall survival; VGPR = very good partial response

The ORR, as reported by participating physicians, was 84.7% (60% ≥ very good partial response, VGPR, 24.7% ≥ complete response, CR). Obtaining a VGPR or better was not influenced by median time of len exposure (*p* = 0.11), starting dose of len (*p* = 0.07), dose of len at time of disease progression (PD, *p* = 0.53), previous exposure to bortezomib (*p* = 0.63) or carfilzomib (*p* = 0.73). The median time to obtain the best response was 3 months. At the time of the analysis, 28 patients were still receiving DaraVd. The treated patients received a median of 9 cycles (IQR 6–27 cycles), and the median duration of treatment was 10 months (IQR 4-25 months). The main cause of discontinuation was PD (n = 48), followed by toxicities (n = 3), death (n = 3), ASCT (n = 1), or unknown reasons (n = 2). With a median follow-up of 22.8 months, median PFS and OS were 14 and 47 months, respectively. Median TTNT was 20 months, while median PFS2, evaluated in 45 patients, was 5 months. PFS and OS curves are shown in Figs. 1 and 2.

In univariate analysis, PFS was positively influenced by obtaining a VGPR or better (hazard ratio, HR = 0.26, 95% CI 0.15–0.46, *p* < 0.001), age ≥ 65 years at relapse (HR = 0.47, 95% CI 0.28–0.80, *p* = 0.005), reduced dexamethasone dose to 20 mg/week (HR = 0.21, 95% CI 0.04–0.96, *p* = 0.045), and the absence of EMD at relapse (HR = 0.28, 95% CI 0.11–0.71, *p* = 0.008). The absence of del17p at relapse was associated with a trend toward a better outcome, though not statistically significant (HR = 0.38, 95% CI 0.14–1.02 *p* = 0.055), the same for a medullary plasma cells (PC) infiltration < 60% (HR = 0.40, 95% CI 0.15–1.05 *p* = 0.062). The presence of standard cytogenetic risk at diagnosis was correlated with better PFS (HR = 0.54, 95% CI 0.29–1.01, *p* = 0.054); however, considering the low number of evaluable patients, this was not confirmed at relapse (HR = 0.90, 95% CI 0.35–2.29, *p* = 0.81). No correlation was found with the duration of exposure to len (*p* = 0.16) and the dose of len at the start of therapy (*p* = 0.49) and at PD (*p* = 0.79).

OS was positively influenced by obtaining a VGPR or better (HR = 0.20, 95% CI 0.1–0.43, *p* < 0.001) age ≥ 65 years at relapse (HR = 0.48, 95% CI 0.24–0.97, *p* = 0.041), dexamethasone 20 mg/week (HR = 0.09, 95% CI 0.02–0.48, *p* = 0.005), 40 mg/week (HR 0.11, 95% CI 0.02–0.51, *p* = 0.005), 80 mg/week (HR 0.17, 95% CI 0.04–0.79, *p* = 0.020), less than 60% of PC infiltration in bone marrow (HR = 0.20, 95% CI 0.057–0.74, *p* = 0.015). The absence of EMD resulted to be not statistically significant (HR = 0.37, 95% CI 0.13–1.08, *p* = 0.068). The absence of high cytogenetic risk at diagnosis or at relapse did not demonstrate to be statistically associated with superior OS. No correlation was found with the duration of exposure to len (*p* = 0.06) and the dose of len at the start of therapy (*p* = 0.40) and at PD (*p* = 0.31).

For PFS2, at univariate analysis, no significant correlation was found. Particularly, no correlation was found with median time to exposure to len, median time to exposure to DaraVd, nor response to DaraVd.

The same characteristics were selected for the building of a multivariate model for PFS and OS: best response VGPR or better, the absence of EMD at relapse and age ≥ 65 years at relapse. Overall, ≥ VGPR and age ≥ 65 years at relapse resulted to be independently associated with improved PFS (respectively, HR = 0.22, 95% CI 0.11–0.42, *p* < 0.001, and HR = 0.49, 95% CI 0.26–0.93, *p* = 0.030). For OS, only best response ≥ VGPR remained statistically significant in the multivariate model (HR = 0.15, 95% CI 0.06–0.38, *p* < 0.001). Univariate and multivariate analysis are shown in Tables 2, 3, 4, and 5.

**Table 2:** Univariate analysis of factors influencing PFS

Characteristics	HR	95% CI	*P* value
Best response ≥ VGPR	0.26	0.15–0.46	< 0.001
Age ≥ 65 years at relapse	0.47	0.28–0.80	0.005
Dexamethasone 20 mg/week	0.21	0.04–0.96	0.045
Absence of EMD at relapse	0.28	0.11–0.71	0.008
Standard cytogenetic risk at diagnosis	0.54	0.29–1.01	0.054
Absence of del17p at relapse	0.38	0.14–1.02	0.055
Medullary plasma cells < 60% at relapse	0.40	0.15–1.05	0.062

Abbreviation: CI = confidence Interval; del = deletion; EMD, extramedullary disease; PFS = progression free survival; VGPR = very good partial response

**Table 3:** Univariate analysis of factors influencing OS

Characteristics	HR	95% CI	*P* value
Best response ≥ VGPR	0.20	0.1–0.43	< 0.001
Age ≥ 65 years at relapse	0.48	0.24–0.97	0.041
Dexamethasone 20 mg/week	0.09	0.02–0.48	0.005
Dexamethasone 40 mg/week	0.11	0.02–0.51	0.005
Dexamethasone 80 mg/week	0.17	0.04–0.79	0.020
Absence of EMD at relapse	0.37	0.13–1.08	0.068
Medullary plasma cells < 60% at relapse	0.20	0.057–0.74	0.015

Abbreviation: CI = confidence Interval; EMD, extramedullary disease; HR = hazard ratio; OS = overall survival; VGPR = very good partial response

**Table 4:** Multivariate analysis for PFS

Characteristics	HR	95% CI	*P* value
Best response ≥ VGPR	0.22	0.11–0.42	< 0.001
Absence of EMD at relapse	0.42	0.15–1.16	0.094
Age ≥ 65 years at relapse	0.49	0.26–0.93	0.030

Abbreviation: CI = confidence Interval; EMD, extramedullary disease; HR = hazard ratio; PFS = progression-free survival; VGPR = very good partial response

Given the evidence of the importance of obtaining a VGPR or better in the univariate analysis, we evaluated the outcome of patients that achieved a response ≥ or < of VGPR. Collectively, mPFS was 26 months in patients achieving at least a VGPR versus 7 months in those achieving inferior responses, while mOS was not reached versus 17 months, respectively. PFS and OS curves stratified by response are shown in Figs. 1 and 2.

### Toxicity

During the study, 58 (68%) patients received all the scheduled doses of daratumumab, while 17 (20%), 4 (5%), and 6 (7%) patients had to skip 1, 2, and 3–5 doses, respectively, mainly due to infections (mostly Covid-19). Bortezomib dose was reduced for thrombocytopenia or PN in 39 (46%) patients, in 62% of cases in the first 3 cycles, and 25 (29%) patients switched to mono weekly administration.

At least one AE of any grade was experimented in 91% (n = 77) of patients, being at least grade ≥ 2 in 85% (n = 72) patients. Infusion-related reaction (IRR) rate with daratumumab was 13% (n = 11): Among reactions, 5 were of grade-1 and 6 of grade-2. Premedication with montelukast was not performed in 6 patients, and two of them developed IRR. Overall, the most common toxicities were PN, infections, asthenia, and cytopenia. Forty-six (54%) patients experimented PN, mostly of grade-1–2, with only 6 (7%) of grade-3, successfully improved or resolved, after bortezomib dose reduction or termination by treatment schedule in 15 (33%) and 21 (46%) patients, respectively. The infection rate was 42% (n = 36), with grade-3 or higher in 15% (n = 13) of cases. Specifically, 11 (13%) patients experienced pneumoniae (grade ≥ 3 in 6 patients), and one patient presented a grade-2 herpes zoster reactivation during antiviral prophylaxis. Hematologic AEs included anemia in 48 (57%) patients, thrombocytopenia in 44 (52%), and neutropenia in 23 (27%), being grade ≥ 3 in 5 (6%), 27 (32%), and 6 (7%), respectively. In addition, asthenia was reported in 31 (37%) patients, while other frequent AEs were gastrointestinal, including diarrhea (n = 19, 22%), nausea (n = 9, 11%), and constipation (n = 16, 19%). Seven patients (8%) experienced skin rash. No patient discontinued treatment for toxicities, but AEs caused death in two patients (one infection and one pneumoniae). Collectively, toxicities are summarized in Table 6.

**Table 5:** Multivariate analysis for OS

Characteristics	HR	95% CI	*P* value
Best response ≥ VGPR	0.15	0.06–0.38	< 0.001
Absence of EMD at relapse	0.94	0.28–3.15	0.918
Age ≥ 65 years at relapse	0.60	0.26–1.40	0.242

Abbreviation: CI = confidence Interval; EMD, extramedullary disease; HR = hazard ratio; OS = overall survival; VGPR = very good partial response

**Table 6:** Toxicity

Characteristics	Number (%)
Anemia	48 (57)
G1	28 (33)
G2	15 (18)
G3/4	5 (6)
Thrombocytopenia	44 (52)
G1	5 (6)
G2	12 (14)
G3/4	27 (32)
Neutropenia	23 (27)
G1	7 (8)
G2	10 (12)
G3/4	6 (7)
Infusion-related reaction	11 (13)
G1	4 (5)
G2	6 (7)
Peripheral neuropathy	46 (54)
G1	14 (17)
G2	26 (31)
G3/4	6 (7)
Resolved	15 (33)
Improved	21 (46)
Infection	36 (42)
G1	6 (7)
G2	18 (21)
G3/4	13 (15)
G5	2 (2)
Pneumoniae	11 (13)
G1	1 (1)
G2	4 (5)
G3	6 (7)
G5	1 (1)
Herpes zoster reactivation	1 (1)
G2	1 (1)
Asthenia	31 (37)
G1	18 (21)
G2	9 (11)
G3/4	4 (5)
Diarrhoea	19 (22)
G1	11 (13)
G2	7 (8)
G3/4	1 (1)
Nausea	9 (11)
G1	8 (9)
G2	1 (1)
Constipation	16 (19)
G1	12 (14)
G2	4 (5)
Skin rash	7 (8)
G1	1 (1)
G2	6 (7)
Hepatic toxicity	3 (3)
G1	1 (1)
G2	1 (1)
G3/4	1 (1)

Abbreviation: G = grade

## Discussion and conclusions

The recent evolution in MM therapy has led to an increase in the use of frontline combination therapies based on anti-CD38 antibodies and len administered until disease progression, both in elderly patients, as supported by the MAIA [33], CEPHEUS [34], and IMROZ [35] trials, and in post-ASCT maintenance settings, as shown in the PERSEUS [36] and AURIGA [37] studies. As a consequence, the majority of patients are len-ref at first relapse, thereby limiting choices for subsequent treatments.

At present, the EMA approved combinations in len-ref patients include DaraVd, PVd, SelVd, IsaKd, DaraKd, DaraPd, and cilta-cel. BelaVd and BelaPd received a positive opinion from CHMP. Importantly, the choice of treatment in patients who are refractory to both len and anti-CD38-containing regimens is even more challenging. In such cases, second-line regimens containing daratumumab or isatuximab are expected to be used less frequently and will remain viable options only in selected patients who discontinued daratumumab as per the treatment schedule outlined in the PERSEUS study [36]. Therefore, therapeutic alternatives for double-refractory patients become significantly limited. Overall, the choice of the best therapy is driven by several factors, including efficacy, expected toxicities, patient age and fitness, and logistical challenges. Moreover, and most importantly, potential therapeutic sequencing must be considered, to avoid limiting possible choices in subsequent lines of therapy. In this regard, third line-approved therapies are pomalidomide-based (elotuzumab, pomalidomide, and dexamethasone) or carfilzomib-based (carfilzomib and dexamethasone) combinations. Therefore, any refractoriness acquired during the second line of treatment may prevent the use of these important therapeutic agents in the following lines of therapy. In later lines of therapy, treatment choices—always to be considered in relation to the refractoriness profile acquired during previous regimens—primarily include idecabtagene vicleucel [38] from the third line onward, and bispecific antibodies such as teclistamab [39], elranatamab [40], linvoseltamab [23], and talquetamab [24] from the fourth line.

In the current treatment scenario, cilta-cel represents the most promising option, but related toxicities and its availability make it a niche product, relegated to young and fit candidates, to be referred in hub centers for its administration. Among triplet combinations, the most promising results have been shown by IsaKd in the IKEMA trial, with a mPFS of 35.7 months and an ORR of 88% and DaraKd in CANDOR, with a mPFS of 28.4 months and an ORR of 85% [10, 15]. Therefore, IsaKd/DaraKd currently represent the main choices at first relapse for patients who are not eligible to receive cilta-cel. However, the major limitations in the use of IsaKd are represented by the associated cardiovascular toxicity, along with the logistics of frequent visits to the hospital, due to the treatment schedule and the intravenous formulation.

Notably, despite the several therapeutic options currently available, few trials enrolled len-exposed and len-ref patients. More importantly, the population of len-ref patients at first relapse was specifically analyzed in the OPTIMISMM trial only, resulting in a PFS of 17.8 months for the PVd arm. On the contrary, only a minority of patients was len-ref in the CASTOR 20, IKEMA [15], CANDOR [10], and BOSTON [16, 25] trials (24%, 32%, 32%, and 27%, respectively), and no data about the efficacy of DaraVd, IsaKd, and SelVd combinations in patients who were refractory to len at first relapse were reported. Similarly, though 79% of the patients enrolled in the DaraPd arm in the APOLLO [14, 26] trial were len-ref, only 11% of them were enrolled at first relapse, and data in this particular setting are not available. Further data on DaraPd from the MM-014 [27] phase II trial were collected in less pre-treated patients (1 median prior line of therapy) but with a consistent percentage (75%) of len-ref patients, 58% of patients len-ref at first relapse, showing a general PFS of 23.7 months in len-ref group. All patients enrolled in the CARTITUDE-4 were len-ref, but only 32.7% of them was at first relapse and data in that specific subgroup are lacking [17]. Of note, even less data are currently available on double-refractory patients. Indeed, only the registration trials of cilta-cel [17] and the combination of belantamab mafodotin plus pomalidomide and dexamethasone [19] included patients with these characteristics, whereas such patients were excluded from the pivotal trials evaluating BelaVd [18], SelVd [16], and PVd [13].

With the limits due to the retrospective design of the study, the aim of the present analysis was to focus on the efficacy of DaraVd triplet as a second-line therapy for len-ref patients in a real-life setting. Indeed, despite this population is increasingly growing in clinical practice, it is poorly represented in the main clinical trials, while representing the main application of DaraVd in real life. Collectively, our data showed a mPFS of 14 months, approximately twice compared to that of len-ref patients included in CASTOR (7.8 months) [20] regardless of the line of therapy, and shorter than mPFS at first relapse in the whole CASTOR study population (27 months), and comparable with the 17.8 months recorded in OPTIMISMM study [13]. The median OS in our patients resulted to be 47 months, thereby improving the mOS of 29 months registered in the immunomodulatory refractory patients in the CASTOR trial [28]. No OS data are available for the other triplets in len-ref patients at first relapse. In the MM-014 trial, the median OS with DaraPd in len-ref patients was 53.6 months [27]. The survival data observed in our retrospective analysis compare favorably with the mPFS of 14.8 months and mOS of 28.9 months reported in a collection of len-ref patients treated with daratumumab-containing combination in clinical trials [29].

Moreover, the possibility of an impact of len duration and dose on the efficacy of DaraVd at relapse was explored; however, no correlation was found in the univariate analysis, consistent with retrospective data reported by Goel and colleagues about second-line treatment of len-ref patients [30]. Conversely, according to our data, the achievement of a VGPR or better was found to be strictly correlated with better PFS and OS, confirmed both in the univariate and multivariate analyses. Indeed, mPFS in patients achieving at least a VGPR was 26 months, being comparable with the PFS obtained in patients treated in second line of therapy in the CASTOR study, independently of the refractoriness status (27 months) [20], and with the PFS obtained with DaraPd in len-ref patients in the MM-014 trial [27]. Moreover, at a median follow-up of 22.8 months, the median OS was significantly better in patients that achieved a VGPR or better (not reached versus 17 months, HR = 0.15, *p* < 0.001). Noteworthy, with electrophoresis and serum immunofixation, largely used in clinical practice, it is not possible to discriminate between daratumumab and monoclonal IgG Kappa isotype M protein. Likely, this is the most probably causes of the high prevalence of VGPR over CR, and of the positive correlation of VGPR with PFS and OS in our study.

Although other real-life retrospective experiences investigated DaraVd, no one focused on len-ref patients at first relapse. In particular, one Italian study conducted by Barilà and colleagues enrolled 57 len-exposed patients with a 77% of patients len-ref and a median of 2 prior lines (a population similar to that of the OPTIMISMM trial), showing a median PFS of 16 months in len-ref patients [31], consistent with our data.

Collectively, our data confirmed DaraVd combination to be safely applicable in real-life settings, with a toxicity profile comparable to what expected, particularly in terms of PN, infections, and thrombocytopenia. Of note, comparing toxicity data with those resulting from the use of IsaKd, while carfilzomib was burdened by a characteristic cardiac toxicity, isatuximab was associated with far more IRR and overall more infections (especially pneumoniae) [15]. In addition, IsaKd is characterized by an intense schedule with two drugs to be administered IV, which makes compliance difficult, especially in elderly patients. On the contrary, the DaraVd combination has shown to be a reliable option, that can be safely and efficaciously used even in older patients, as almost half of our study population was over 70 years old; moreover, in multivariate model, age ≥ 65 was not correlated with inferior outcomes in terms of PFS and OS. In addition, in our univariate analysis, dexamethasone dose reduction was not correlated with inferior OS, supporting the possibility of widespread use of DaraVd in the elderly population or in patients with comorbidities, such as diabetes. Regarding PFS, the 27 months mPFS obtained by patients who underwent ASCT was inferior compared to that of 52.8 months reported in the meta-analysis performed by McCarthy and colleagues [32], likely due to the different inclusion criteria of the present analysis with respect to those of patients included in the published data.

Certainly, our study harbors some limitations, the major being the retrospective design of the study and the small number of patients included in the analysis—that hampered the possibility of subgroup analyses—the little availability of FISH analyses at relapse, and nearly the absence of patients older than 80 years of age.

In conclusion, despite the rapidly evolving treatment scenario of RRMM and the progressive availability of newer treatments, including T-cell redirecting therapies in earlier lines, DaraVd demonstrated to be a good option for len-ref patients at first relapse, especially for elderly, frail, or those ineligible to receive more intensive/toxic regimens. Not least, given the limited accessibility to more advanced immunotherapies based on chimeric antigen receptor T-cells in non-accredited centers, as well as the limited availability of resources in different districts, DaraVd may also represent a valid cost-effective alternative, with an ease of route and schedule of administration that may facilitate treatment adherence in these patients.

## Data Availability

The data supporting the findings of this study are available via Zenodo platform (10.5281/zenodo.16091434).
